# Effects of clenbuterol administration on mitochondrial morphology and its regulatory proteins in rat skeletal muscle

**DOI:** 10.14814/phy2.14266

**Published:** 2019-10-09

**Authors:** Yu Kitaoka, Daiki Watanabe, Yudai Nonaka, Kazuyoshi Yagishita, Yutaka Kano, Daisuke Hoshino

**Affiliations:** ^1^ Department of Human Sciences Kanagawa University Yokohama Japan; ^2^ Graduate School of Integrated Arts and Sciences Hiroshima University Hiroshima Japan; ^3^ Department of Engineering Science The University of Electro‐communications Chofu Tokyo Japan; ^4^ Clinical Center for Sports Medicine and Sports Dentistry Hyperbaric Medical Center/Sports Medicine Clinical Center Medical Hospital of Tokyo Medical and Dental University Bunkyo‐ku Tokyo Japan

**Keywords:** β2 adrenergic agonist, muscle fiber type, mitochondrial dynamics

## Abstract

Clenbuterol induces a slow‐to‐fast fiber type transition in skeletal muscle. This muscle fiber transition decreased mitochondrial oxidative capacity and respiratory function. We hypothesized that the clenbuterol‐mediated reduction in oxidative capacity is associated with the alteration in mitochondrial morphology. To verify this hypothesis, we examined whether clenbuterol alters mitochondrial morphology and mitochondrial regulatory proteins in rat skeletal muscle. Clenbuterol was administered to rats via drinking water (30 mg/L) for 3 weeks. Myosin heavy chain (MHC) isoform composition, mitochondrial morphology, and fusion and fission regulatory protein levels in deep region and superficial region in tibialis anterior (TA) muscles were assessed. Clenbuterol induced the fiber type transition from slow to fast in both the regions of TA. The levels of optic atrophy protein 1, mitofusin 2, and mitochondrial fission 1, but not of dynamin‐related protein 1, significantly decreased in deep and superficial muscles after clenbuterol administration (*P* < 0.01). Also, observation using the transmission electron microscopy showed a decrease in mitochondrial volume (*P* < 0.05) and an increase in proportion of continuous or interacting mitochondria across Z‐lines (*P* < 0.05). We showed that clenbuterol administration induces a transition in the muscle fiber type composition toward fast phenotype and causes alterations in mitochondrial morphology with a concomitant decrease in mitochondrial fusion and fission regulatory protein levels. These mitochondrial morphological alterations may influence deleterious effects on skeletal muscle metabolism.

## Introduction

Clenbuterol, a β2‐adrenergic agonist, is commonly prescribed as a bronchodilator for the treatment of asthmatic patients. In addition to its anti‐asthma effect, it causes a significant increase in muscle mass and strength in rodents when administered for long term (Zeman et al., 1988; Dodd et al., 1996). Owing to this anabolic effect, clenbuterol has the potential to ameliorate muscle wasting in pathological conditions (Lynch and Ryall, 2008), and also to be exploited by athletes.

Long‐term clenbuterol administration induces a transition from slow‐to‐fast muscle fiber type composition, which is defined by myosin ATPase activity (Zeman et al., 1988) and myosin heavy chain (MHC) isoforms (Dodd et al., 1996; Kitaura et al., 2001; Oishi et al., 2002; Ohnuki et al., 2016); this transition is accompanied by increased muscle force and decreased fatigue tolerance (Zeman et al., 1988; Dodd et al., 1996). Muscle metabolic characteristics also change with the transition in the fiber type composition toward fast phenotype (Torgan et al., 1993; Dodd et al., 1996; Hoshino et al., 2012). We have previously reported that the levels of markers of mitochondrial content and respiratory function decrease with clenbuterol administration, indicating its deleterious effects on skeletal muscle metabolism (Hoshino et al., 2012).

Mitochondria are organized in a morphologically plastic network regulated by fusion and fission; these processes, termed mitochondrial dynamics, play a crucial role in the maintenance of functional mitochondria. The expression of mitochondrial dynamics proteins correlates with the oxidative capacity of muscle fibers (Iqbal et al., 2013). Recent evidence has shown that endurance exercise training leads to a more fused, elongated mitochondrial network, with muscle fiber type transition toward slow phenotype (Iqbal et al., 2013; Axelrod et al., 2018). However, it remains unclear whether clenbuterol, which induces slow‐to‐fast muscle fiber type transition, can alter mitochondrial morphology and the expression of dynamics regulatory proteins. We hypothesized that the clenbuterol‐mediated reduction in oxidative capacity is not only attributable to muscle mitochondrial content reduction but also associated with concurrent changes in mitochondrial morphology. In this study, we first assessed proteins involved in oxidative phosphorylation (OXPHOS) as markers of mitochondrial content. Next, we examined whether clenbuterol administration affects protein levels involved in mitochondrial fusion and fission events. Furthermore, transmission electron microscopy analysis allowed us to assess changes in mitochondrial morphology in response to clenbuterol administration.

## Methods

### Animals and clenbuterol administration

We purchased 12 Wistar male rats (aged 8 weeks) from Japan SLC Inc. (Shizuoka, Japan). The rats were housed on a 12:12‐h light‐dark cycle in an air‐conditioned room. Following a 3‐ to 7‐day acclimation period, the animals were randomly divided into two treatment groups: control group (*n* = 6), which was provided with standard chow and water *ad libitum* for 3 weeks, and clenbuterol group (*n* = 6), which was provided with standard chow and treated for 3 weeks with clenbuterol (Sigma‐Aldrich, St. Louis, MO, USA) administered via drinking water (30 mg/L), as previously reported (Stevens et al., 2000; Oishi et al., 2002; Hoshino et al., 2012). Two rats were housed in a cage (22 cm × 38 cm with 20 cm height). Body weight and amount of water intake were recorded 3 times a week. Water intake per two animals in a cage was not significantly different between the two groups during the experimental period. After 3 weeks, the animals were anesthetized (65 mg of pentobarbital sodium/100 g body weight), and tibialis anterior (TA) muscles were excised. We separated into deep and superficial region for performing immunohistochemical staining and western blotting because we examined mitochondrial markers and mitochondrial respiratory function in these regions of TA in the previous study (Hoshino et al., 2012). Separation of the deep and superficial TA compartments was based on their distinct anatomical locations in the muscle of control animals. This separation is a common method to examine the effects of pharmacological and physiological stimulus on metabolic adaptation in different fiber type of rat skeletal muscles (Bonen et al., 2000; Enoki et al., 2006; Benton et al., 2008). Muscle samples were stored at −80°C until further immunohistochemical and western blot analyses. The treatment procedures were approved by the Institutional Animal Care and Use Committee of University of Electro‐Communications (Tokyo, Japan; Approval No. 29).

### Immunohistochemical staining

The fiber type composition of MHCI, IIa, and IIb was determined through immunohistochemical staining as described previously (Watanabe et al., 2017). Briefly, the excised TA muscle blocks were rapidly frozen in isopentane and cooled in liquid nitrogen. Serial 10‐μm sections were made with a cryostat (CM1950; Leica Biosystems, Jena, Germany) at −20°C and mounted on polylysine‐coated slides. MHC monoclonal antibodies obtained from Developmental Studies Hybridoma Bank (Iowa city, IA, USA) were used (dilution, 1:100; BA‐F8 for MHCI, SC‐71 for IIa, BF‐F3 for IIb). The sections were allowed to be warmed at room temperature and were incubated in phosphate‐buffered saline (PBS) (pH 7.5) at 25°C before further incubation with a primary antibody in a humidified box overnight at 4°C. Vectastain ABC kit (Burlingame, CA, USA) was used according to manufacturer’s instructions to reveal the immunohistochemical reaction. Muscle fibers stained by each MHC isoform were counted within total 100 muscle fibers. The images were subsequently analyzed using ImageJ software.

### Western blot analysis

Samples were homogenized in radioimmunoprecipitation assay buffer (25 mmol/L Tris‐HCl, pH 7.6, 150 mmol/L NaCl, 1% NP‐40, 1% sodium deoxycholate, and 0.1% sodium dodecyl sulfate [SDS]) supplemented with protease inhibitor mixture (Complete Mini, ETDA‐free, Roche Applied Science, Indianapolis, IN) and phosphatase inhibitor mixture (PhosSTOP; Roche Applied Science, Penzberg, Germany). The total protein content of the samples was quantified using the bicinchoninic acid assay (Pierce Biotechnology, Rockford, IL, USA). Equal amounts of protein were loaded onto 10% SDS‐PAGE gels and separated through electrophoresis. Proteins were transferred to polyvinylidene difluoride membranes, and then western blotting was performed using primary antibody of Total OXPHOS Rodent WB Antibody Cocktail (ab110413), mitofusin 2 (Mfn2, ab124773), dynamin‐related protein 1 (Drp1, ab56788), and mitochondrial fission 1 (Fis1, ab96764) purchased from Abcam (Cambridge, UK), in addition to optic atrophy protein 1 (Opa1, #612606) prepared from BD Transduction Laboratories (BD Biosciences, San Jose, CA, USA). The Ponceau staining method was used to verify consistent loading and appropriate transfer for each blot. Blots were then scanned and quantified using a C‐Digit Blot Scanner (LI‐COR Biosciences, Lincoln, NE, USA).

### Transmission electron microscopy

Muscle bundles (~10 fibers) were fixed with 2% glutaraldehyde and 2.5% formaldehyde in 0.1 M phosphate buffer (PB) solution (pH 7.4) for 24 h at 4°C and subsequently washed in 0.1 M PB for 12 h at 4°C. Thereafter, the bundles were post‐fixed with 1% osmium tetroxide in 0.1 M PB for 2 h at 4°C with gentle shaking and then dehydrated through a graded series of ethanol at 25°C. After dehydration, the bundles were infiltrated with a graded mixture of propylene oxide and resin at 25°C and then embedded in longitudinal orientation on 100% resin at 60°C for 48 h. Afterward, 1‐μm thick sections were made to check the orientation of the embedded bundle and section quality. Ultrathin sections were cut using a diamond knife on an ultramicrotome, and the sections were mounted on Pioloform filmed copper grids. After staining with uranyl acetate and lead citrate, the sections were photographed under a transmission electron microscope (TEM) using a charge‐coupled device camera. From each fiber, 3–5 images were obtained at ×30,700 magnification from central regions of the myofibrillar space.

### Stereological methods

Mitochondrial volume was estimated using a standard stereological method (i.e., counting intersections) reported by Nielsen et al. (2010). The mitochondrial volume density (*V*_v_) was estimated using the following formula: *V*_v_ = *A*_A_, where *A*_A_ denotes the mitochondrial area estimated as follows: 420 × 420‐nm grids were superimposed on each TEM image, and then, the number of points that touched mitochondria were tallied and divided by total number of points on the grid (i.e., 462 points in this study). In addition, 4–6 images were taken per fiber, and the images were then analyzed by three investigators. The coefficients of error of mitochondrial volume were as follows: 0.17 (deep region of TA in the control group), 0.09 (superficial region of TA in the control group), 0.11 (deep region of TA in the clenbuterol group), and 0.09 (superficial region of the TA in clenbuterol group).

### Mitochondrial membrane interactions

Intracellular mitochondria interact with neighboring mitochondria, and their reticulum constantly undergoes fusion and fission. Typically, in the longitudinal section, a pair of mitochondria can be observed on both sides of the Z‐line (e.g., arrows in Fig. 3A). However, some pairs of mitochondria interact across Z‐line, or a single mitochondrion spans Z‐line. Using TEM micrographs, the number of Z‐lines possessing mitochondria on both sides (Z‐line_total_) and the number of Z‐lines where a pair of mitochondria spanned (Z‐line_spanned_) were counted. The proportion of Z‐lines spanned by a continuous mitochondrion or interacting mitochondria (Z‐line_spanned_/Z‐line_total_) in each fiber was measured as reported by Picard et al. (2013). The coefficients of error of Z‐line_spanned_/Z‐line_total_ were as follows: 0.13 (deep region of TA in the control group), 0.09 (superficial region of TA in the control group), 0.10 (deep region of TA in the clenbuterol group), and 0.06 (superficial region of TA in the clenbuterol group). Eventually, 431 and 632 pairs of mitochondria in superficial and deep TA, respectively, were analyzed.

### Statistical analysis

All data were presented as mean ± standard error of the mean. Two‐way repeated measures analysis of variance (ANOVA) was used to analyze the differences for the body weights. A two‐way ANOVA was also used to analyze the between‐group differences (control vs. clenbuterol) for muscles (deep vs. superficial). Besides, post hoc comparisons were performed using the Sidak procedure. All statistical analyses were performed using GraphPad Prism version 8.0 Software (GraphPad, San Diego, CA, USA). *P*‐value < 0.05 was considered significant.

## Results

### Body and muscle weights

Body weight was not significantly different between the control and clenbuterol groups before (control: 213.5 ± 3.90 g, clenbuterol: 209.5 ± 4.71 g) and after 3 weeks (control: 280.3 ± 4.59 g, clenbuterol: 287.3 ± 3.30). However, TA muscle weight (control: 0.482 ± 0.01 g, clenbuterol: 0.585 ± 0.01 g) and relative muscle weight (control: 1.84 ± 0.04 mg/g body weight, clenbuterol: 2.24 ± 0.04 mg/g body weight) was significantly higher in the clenbuterol group than in the control group (P < 0.05).

### Muscle fiber type composition.

**Figure 1:**
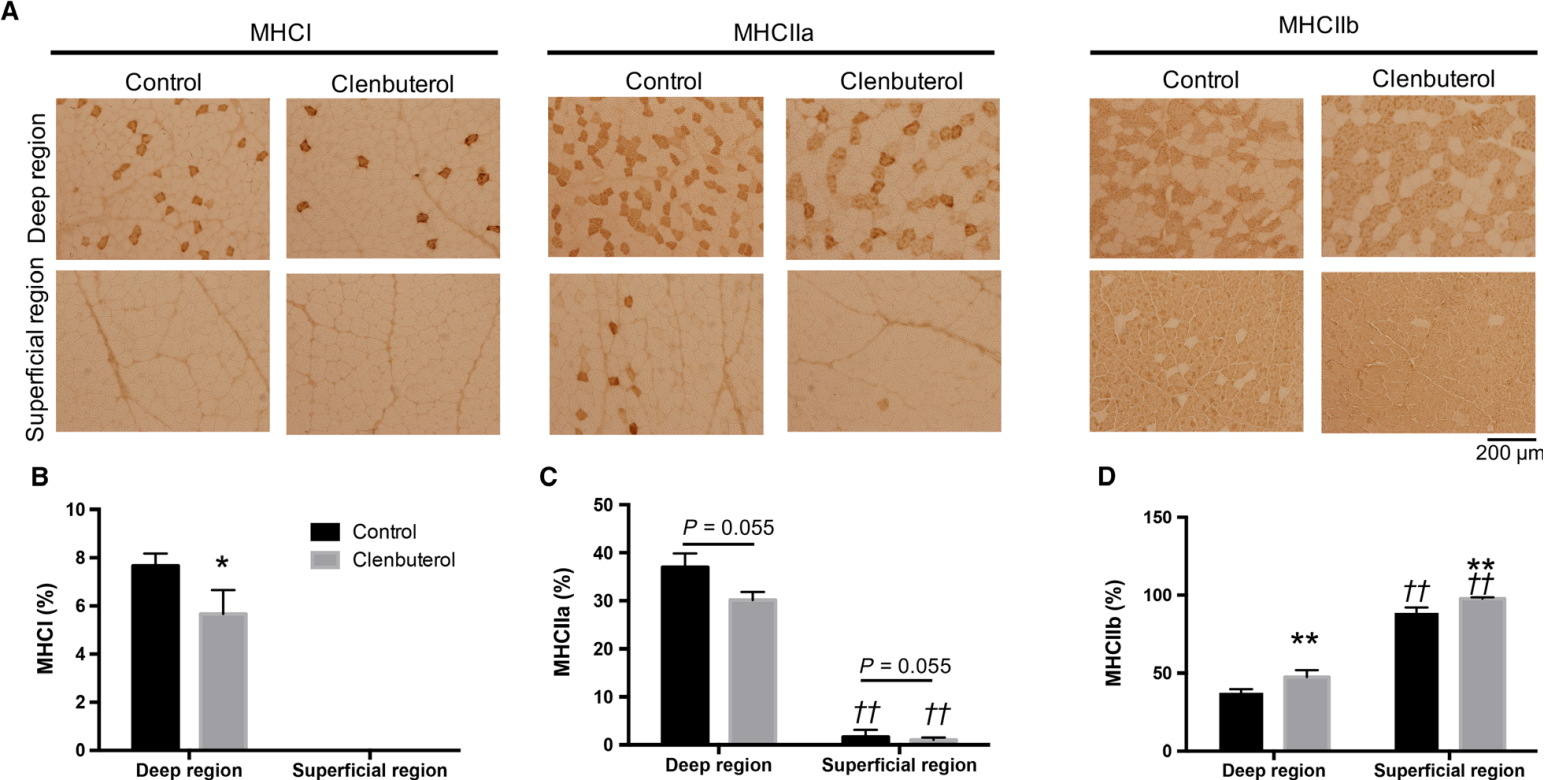
Effects of clenbuterol administration on myosin heavy chain (MHC)I, IIa, and IIb composition. Representative micrographs of imunohistochemical staining for MHCI, MHCIIa, and MHCIIb (A). Quantitative comparison of MHCI (B), MHCIIa (C) and MHCIIb (D) composition (%) in deep and superficial regions of the tibialis anterior muscle. Values are expressed as mean ± standard error of mean; *n* = 6 rats. *P < 0.05, **P < 0.01, significant effect on control group vs. clenbuterol group. ^††^ *P* < 0.01, significant effect on superficial region vs. deep region.

Muscle fiber composition of MHCI was significantly lower in the clenbuterol group than in the control group in deep region, whereas MHCI was not detected in superficial region of TA in either group (*P* < 0.05, Fig1A and 1). Muscle fiber composition of MHCIIa tended to be lower in the clenbuterol group (*P* = 0.055, Fig. 1A and 1), whereas that of MHCIIb was significantly higher in the clenbuterol group than in the control group in both deep and superficial regions (*P* < 0.01, Fig. 1A and 1).

### Mitochondrial proteins

**Figure 2:**
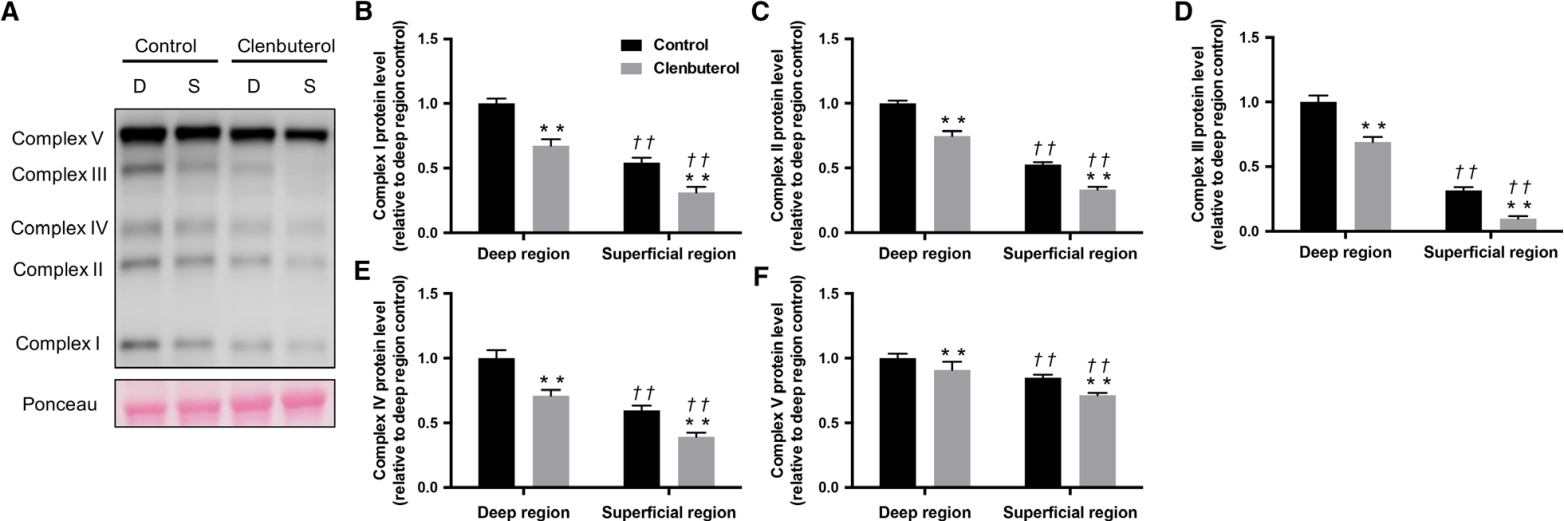
Effects of clenbuterol administration on mitochondria complex protein levels. Representative images of each specific band. Ponceau staining reflected consistent loading and appropriate transfer (A). Mitochondrial complex I (B)*,* complex II (C), complex III (D), complex IV (E), complex V (F) protein levels in deep and superficial regions of tibialis anterior muscles. Values are expressed as mean ± standard error of mean; *n* = 6 rats. **P < 0.01, significant effect on control group vs. clenbuterol group. ^††^ *P* < 0.01, significant effect on superficial region vs. deep region.

**Figure 3:**
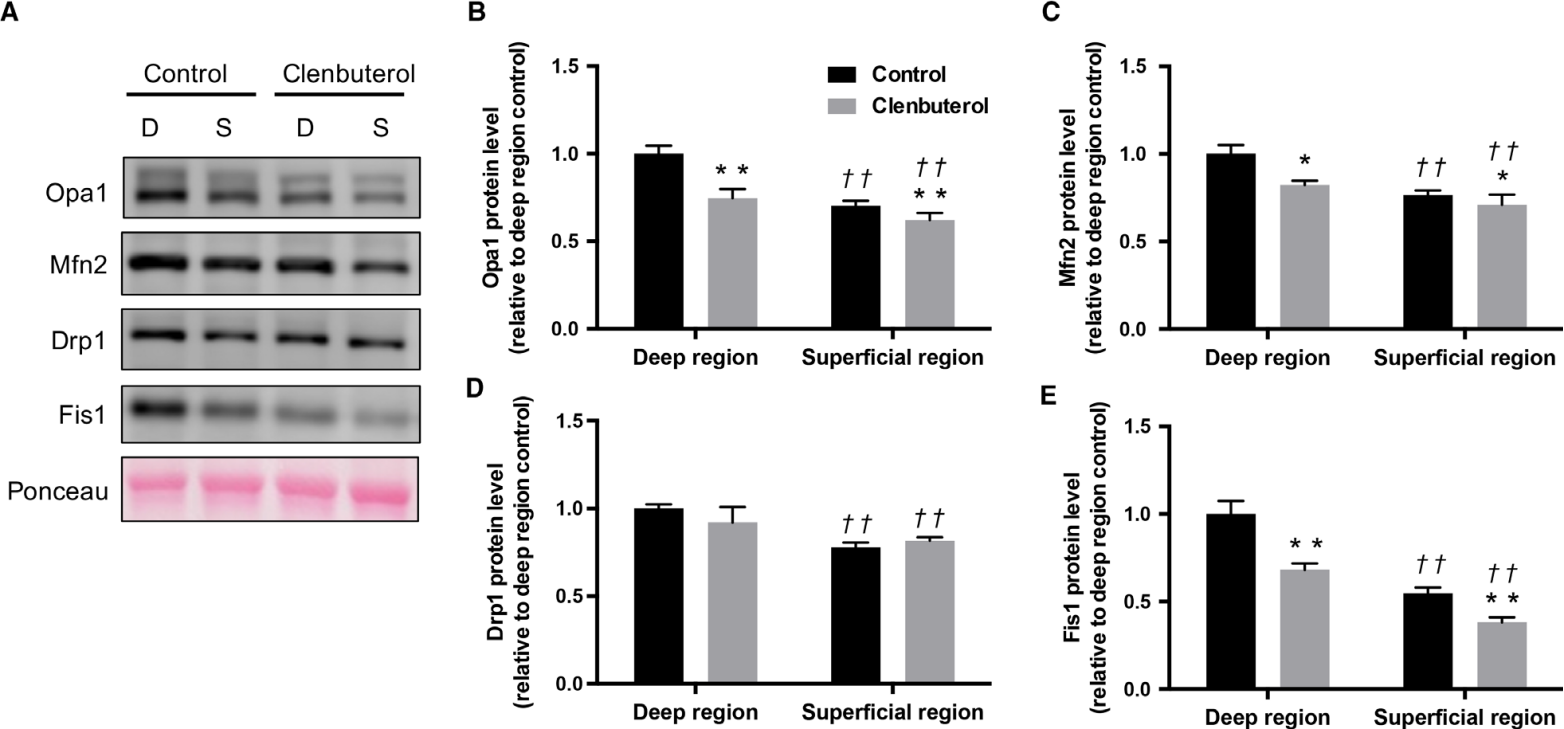
Effects of clenbuterol administration on mitochondrial fusion and fusion protein levels. Representative images of each specific band. Ponceau staining reflected consistent loading and appropriate transfer (A). optic atrophy protein 1 (Opa1) (B), Mfn2 (C) dynamin‐related protein 1 (Drp1) (D), mitochondrial fission 1 (Fis1) (E) protein levels in deep and superficial regions of tibialis anterior muscles. Values are expressed as mean ± standard error of mean; *n* = 6 rats. **P < 0.01, significant effect on control group vs. clenbuterol group. ^††^ *P* < 0.01, significant effect on superficial region vs. deep region.

The levels of all mitochondrial complex (I–V) proteins were significantly lower in the clenbuterol group than in the control group in both deep and superficial muscles (*P* < 0.01, Fig. 2A–F). Additionally, Opa1, Mfn2, and Fis1 levels were significantly lower in the clenbuterol group than in the control group (*P* < 0.05, Fig. 3A–C and E) in both deep and superficial muscles, whereas Drp1 levels remained unaffected (*P* > 0.05, Fig. 3D).

### Mitochondrial volume and morphology

**Figure 4:**
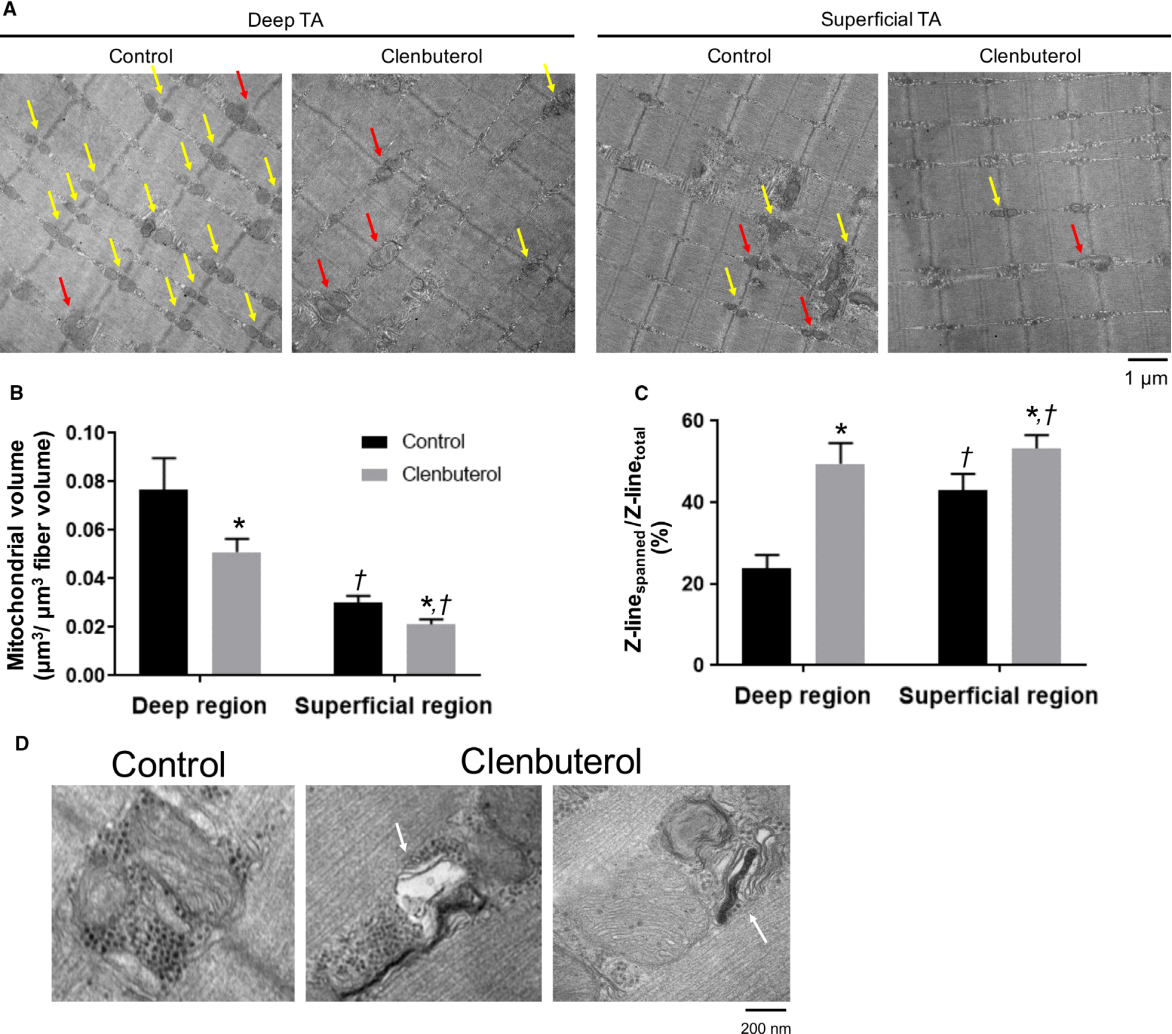
Effect of clenbuterol administration on mitochondrial morphology. Representative micrographs (A). Mitochondrial volume was estimated using standard stereological methods (C). Mitochondria can be found among the myofibrils, with the majority of them aligned with the Z‐line. Some Z‐lines possess mitochondria on both sides (i.e., yellow and red arrows); red arrows indicate continuous or interacting mitochondria across Z‐lines; yellow arrows indicate Z‐line possessing two mitochondria without any interaction. (B). Mitochondria in the clenbuterol group showed disrupted mitochondrial cristae structure (white arrows) (D). Values (mean ± standard error of mean) are average of 9–12 fibers obtained from four rats. *P < 0.05, significant effect on control group vs. clenbuterol group. ^†^ *P* < 0.05, significant effect on superficial region vs. deep region.

The electron micrographs of in the deep and superficial region of TA showed organized fibers with mitochondria in the both groups (Fig. 4A). Mitochondrial volume was 0.077 ± 0.013 μm^3^/μm^3^ fiber volume (deep region of TA in the control group), 0.051 ± 0.005 μm^3^/μm^3^ fiber volume (deep region of TA in the clenbuterol group), 0.030 ± 0.003 μm^3^/μm^3^ fiber volume (superficial region of TA in the control group), and 0.021 ± 0.002 μm^3^/μm^3^ fiber volume (superficial region of TA in the clenbuterol group). Mitochondrial volume was significantly lower in the clenbuterol group than in the control group in both the regions (*P* < 0.05, Fig. 4A and 4). The proportions of continuous or interacting mitochondria across Z‐lines (Z‐line_spanned_/Z‐line_total_) were 23.9% ± 3.2% in the deep region of TA in the control group and 43.1% ± 3.9% in the superficial region of TA in the control group; these values were consistent with previously reported values (Picard et al., 2013). The proportion of mitochondria spanning Z‐line was significantly higher in the clenbuterol group than in the control group in the deep (49.4% ± 5.1%) and superficial (53.3% ± 3.3%) regions (*P* < 0.05, Fig. 4C). Furthermore, mitochondria in the clenbuterol group showed disrupted and abnormal mitochondrial cristae structure, which is classic ultrastructural signs of mitochondrial dysfunction (Fig. 4D).

## Discussion

We examined whether clenbuterol alters mitochondrial morphology and mitochondrial protein levels in deep and superficial region of TA muscles. Along with the fiber type transition from slow to fast, we found that clenbuterol decreased the levels of mitochondrial OXPHOS proteins as well as those of proteins involved in fusion (Mfn2, Opa1) and fission (Fis1). Furthermore, we observed a reduction in the mitochondrial volume and an increase in the proportion of continuous or interacting mitochondria across the Z‐line. These results suggest that clenbuterol‐induced slow‐to‐fast muscle fiber type transition alters mitochondrial dynamics protein and mitochondrial morphology.

The transition in fiber type composition toward fast phenotype was observed in both deep and superficial regions after clenbuterol administration over 3 weeks and was consistent with previous reports (Zeman et al., 1988; Dodd et al., 1996; Kitaura et al., 2001; Oishi et al., 2002; Ohnuki et al., 2016). Accompanied with the slow‐to‐fast fiber type transition, mitochondrial volume analyzed using TEM decreased in both deep and superficial regions following clenbuterol administration. This observation was consistent with western blotting results that showed decreased mitochondrial OXPHOS protein levels. These changes in markers of mitochondrial contents are consistent with decreases in citrate synthase (CS) activity and COX IV protein contents of TA muscles in our previous study (Hoshino et al., 2012). Furthermore, levels of mitochondrial fusion proteins Opa1 and Mfn2 and fission protein Fis1 were decreased after clenbuterol administration. These protein levels also associated with alteration in muscle fiber composition. Denervation decreases mitochondrial volume with down‐regulation of mitochondrial fusion proteins Opa1 and Mfn2 (Iqbal et al., 2013; Kitaoka et al., 2016), whereas chronic contractile activity leads to reticular mitochondria with up‐regulation of these proteins (Iqbal et al., 2013; Kitaoka et al., 2015). These findings suggest that slow‐to‐fast fiber type transition decreased mitochondrial volume and dynamics protein levels. The machinery of mitochondrial fusion and fission is essential for the maintenance of functional mitochondrial network in skeletal muscle. Previous studies have demonstrated that both fusion and fission regulatory proteins are suppressed in sarcopenia and cancer cachexia (Barreto et al., 2016; Tezze et al., 2017). Muscle‐specific ablation of both fusion and fission proteins induces the accumulation of abnormal mitochondria and the inhibition of mitophagy (Favaro et al., 2019; Romanello et al., 2019). This prompted us to examine mitochondrial morphology and membrane interactions by TEM.

Decreased mitochondrial dynamics protein levels should alter mitochondrial network, and, to address this point, the proportion of continuous or interacting mitochondria across Z‐lines (i.e., Z‐line_spanned_/Z‐line_total_) was measured as an indicator of mitochondrial interactions. The interaction between mitochondria across Z‐line is enhanced immediately after exercise (Picard et al., 2013). In contrast, muscle disuse reduces mitochondrial interactions across Z‐lines, associated with mitochondrial dysfunction (Picard et al., 2015). In the present study, clenbuterol increased the Z‐line_spanned_/Z‐line_total_ in both regions of TA, which suggests that clenbuterol enhanced the mitochondrial interactions despite decreased mitochondrial volume. This may be a compensatory attempt to rescue mitochondrial function, since we observed disrupted or swollen mitochondria characterized by few of the densely packed cristae membranes in TEM images of the clenbuterol group. Such abnormal mitochondria are typically observed in skeletal muscles of aged and mitochondrial DNA mutator mice with the impairment of mitochondrial respiration (Safdar et al., 2011; Leduc‐Gaudet et al., 2015). Importantly, we have previously demonstrated that clenbuterol administration impairs mitochondrial fatty acid oxidation and its marker enzymes in isolated mitochondria of both deep and superficial TA muscles (Hoshino et al., 2012). Consequently, clenbuterol‐induced morphological changes observed in the present study (i.e., decreased mitochondrial volume and increased Z‐line_spanned_/Z‐line_total_) may be ascribable to the increased number of abnormal mitochondria characterized by respiratory dysfunction and unstable morphology.

These morphological alterations in mitochondria were paralleled with decreased mitochondrial dynamics protein levels mediated by clenbuterol‐induced muscle fiber type transition. However, the slow‐to‐fast muscle fiber type transition seems not necessarily to induce mitochondrial abnormal ultrastructure, because mitochondria in the superficial region of TA, where the most of fibers are fast‐twitch, were not abnormal if clenbuterol was not administrated. β2‐adrenergic stimulation affects substrate metabolism (Hoshino et al., 2012), Ca^2+^ homeostasis (Cairns and Borrani, 2015), apoptosis (Burniston et al., 2005), and protein synthesis (Kline et al., 2007) in skeletal muscle. Thus, the possibility remains that these various factors, caused by β‐adrenergic stimulation also affect mitochondrial morphology and function in skeletal muscle.

Lastly, we should note the limitation of our approach in this study. We separated TA muscle into the deep and superficial regions as in our previous study (Hoshino et al., 2012), however, it is difficult to separate completely the same region in different animals. Future study is required using different skeletal muscles such as soleus (slow‐twitch) and extensor digitorum longus (fast‐twitch). Another limitation is that we did not analyze the fiber size for each fiber type, which affects metabolic adaptation in skeletal muscle.

In conclusion, we showed that clenbuterol administration induces a transition in the muscle fiber type composition toward fast phenotype and causes alterations in mitochondrial morphology with a concomitant decrease in mitochondrial fusion and fission regulatory protein levels. These results are of great significance for understanding the relationship between transition in muscle fiber type and mitochondrial adaptation.

## Conflicts of Interest

None of the authors has any conflict of interest to disclose.
